# Spare-part management in a heterogeneous environment

**DOI:** 10.1371/journal.pone.0247650

**Published:** 2021-03-19

**Authors:** Reza Barabadi, Mohammad Ataei, Reza Khalokakaie, Ali Nouri Qarahasanlou

**Affiliations:** 1 Faculty of Mining, Petroleum & Geophysics, Shahrood University of Technology, Shahrood, Semnan, Iran; 2 Faculty of Technical & Engineering, Imam Khomeini International University, Qazvin, Iran; Fuzhou University, CHINA

## Abstract

Spare-part management has a significant effect on the productivity of mining equipment. The required number of spare parts can be estimated using failure and repair data collected under the name of reliability data. In the mining industry, failure and repair times are decided by the operational environment, rock properties, and the technical and functional behavior of the system. These conditions are heterogeneous and may change significantly from time to time. Such heterogeneity can change equipment’s reliability performance and, consequently, the required number of spare parts. Hence, it is necessary for effective spare-part planning to check the heterogeneity among the reliability data. After that, if needed, such heterogeneity should be modeled using an adequate statistical model. Heterogeneity can be categorized into observed and unobserved caused by risk factors. Most spare-part estimation studies ignore the effect of heterogeneity, which can lead to unrealistic estimations. In this study, we introduce the application of a frailty model for modeling the effect of observed and unobserved risk factors on the required number of spare parts for mining equipment. Studies indicate that ignoring the effect of unobservable risk factors can cause a significant bias in estimation.

## 1. Introduction

Spare-part estimation plays a crucial role in logistic management. Effective spare-part planning reduces equipment downtime and prevents unnecessary inventory, one of the most critical wastes in the production process. Studies show that spare parts shortage is one of the main reasons for downtime in mining equipment [1,2]. Mining companies often rely on the manufacturer’s recommendations. The required number of spare parts is calculated, mostly based on reliability data, including time between failure (TBF) and time to repair (TTR). However, studies have shown that, in addition to TBF and TTR data, the operational conditions in which the equipment is working should also be considered in spare-part planning [3]. For example, Barabadi et al. [4,5] have shown that ignoring the effect of operating conditions may lead to a 25% difference in the estimated number of spare parts. Operational conditions and other factors that may influence an item’s reliability characteristics are named risk factors (covariates). Risk factors can be categorized as observed and unobserved risk factors. Unobservable risk factors are those factors for which we have no possible way to form a database and perform analysis of their effects as we can for recordable factors (observable risk factors). Recently, the effect of observed risk factors on reliability and the required number of spare parts has been studied. For example, after presenting the reliability analysis algorithm with time data in the context of mining studies, Kumar, in collaboration with Klefsjö [6,7], proposed the Proportional Hazard Model (PHM) to analyze the effect of observable risk factors in reliability analysis. This model was used in later years by various researchers in the field of mining, such as Ghodrati [8–12], Abbas Barabadi [5], and Nouri Qarahasanlou [13–17], to analyze the reliability of mining equipment and finally, the required number of spare parts. However, the PHM or its extensions, such as the Stratified Cox Regression Model (SCRM) can only be used to analyze observable risk factors. Although unobserved risk factors may significantly change the required number of spare parts, most reliability studies have ignored their effect.

Researchers in other fields such as medicine have used the frailty model (Mixed Proportional Hazard Model) to isolate the effect of unobserved risk factors. In this model, all unobservable risk factors and measurement errors are considered a random multiplier phrase and added to the PHM model. Vaupel et al. [18] were the first to use the term ‘frailty’ for univariate survival models. Gutierrez [19] studied frailty and shared frailty models, compare their characteristics, and analyzed the frailty model’s survival data. The frailty models of survival data appear similar to regression models due to the heterogeneity and random effects. Frailty is a hidden multi-effect on the hazard function’s performance, with a mean of 1 and variance of θ, which has been estimated along with the model’s other parameters. Since 2006, some studies on reliability have used the frailty model for modeling the impact of unobservable risk factors on the reliability of the components, intending to make decisions about maintenance and repair [20–23]. For example, Asha [24] employed the frailty model for a load-sharing system and demonstrated that the reliability analysis for a heterogeneous case varies significantly compared to a homogeneous one. Asfaw and Lindqvist [25] utilized the frailty model for modeling the effect of observable and unobservable risk factors on wind turbine reliability, using Poisson’s process. Slimacek and Lindqvist [26] examined the unobservable heterogeneity results in repairable systems by the heterogeneous Poisson’s process (Power-law). The results of their studies show that when there are several similar systems for examination, there will be an unknown heterogeneity between the systems, and ignoring such unobserved heterogeneity may lead to wrong decisions.

Mining operational conditions are not identical, and, in reality, it is a heterogeneous environment in which characteristics change significantly. The frailty model is a suitable model that can be used for molding the effect of unobserved heterogeneity on the required number of spare parts in the mining industry. Despite the frailty model’s suitability, its application is not well developed in reliability engineering. There is no such application for spare-part estimation as far as we know. This paper’s primary motivation is to develop a framework for spare-part estimation, considering the effect of observed and unobserved heterogeneity, using a frailty model. The rest of the paper is organized as follows. In Section 2, the proposed framework is discussed; after that, its application is discussed in Section 3. Section 4 provides conclusions.

## 2. The methodology: Spare-part prediction using frailty model

**Fig 1:**
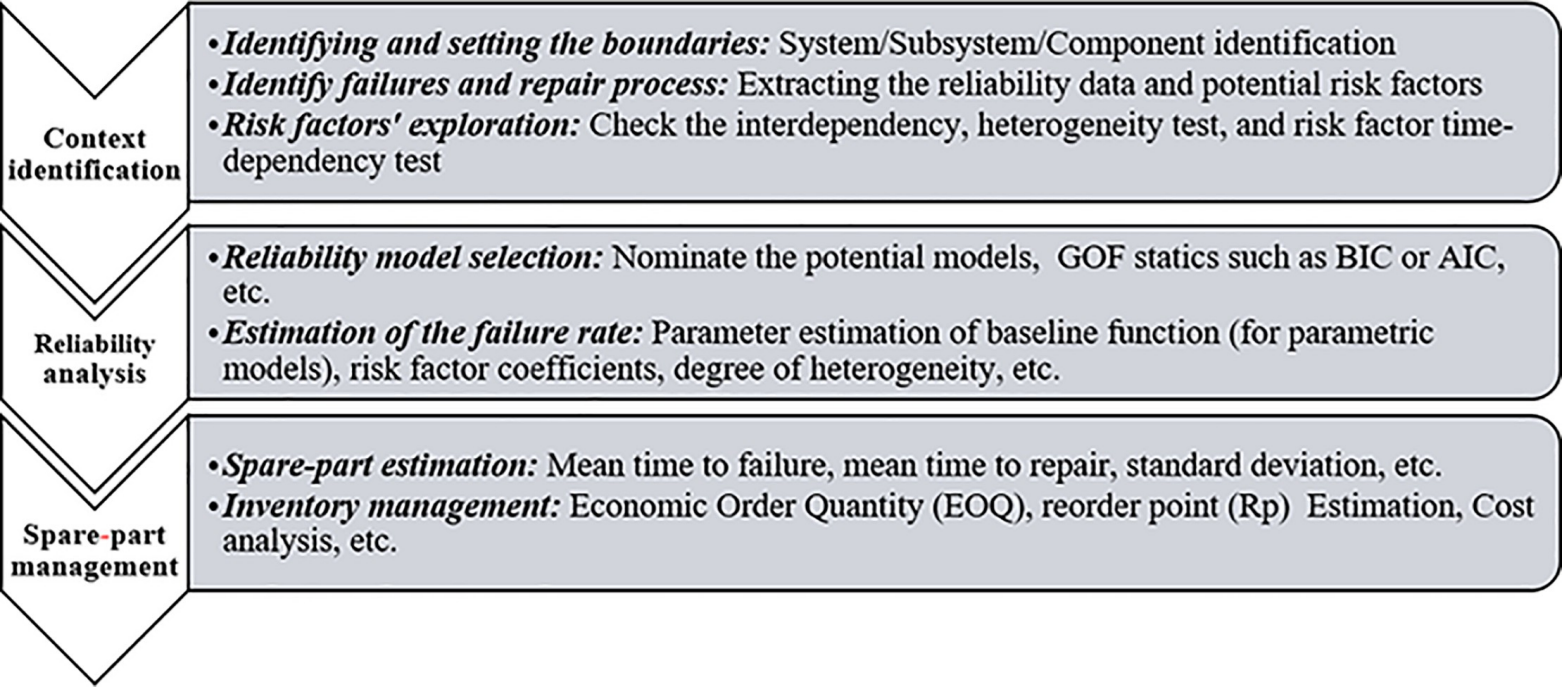
Spare parts based on reliability and risk factors.

Fig 1 shows the framework for spare-part prediction using a frailty model. The framework includes three steps:

Step 1: Context identification

- Item identification and boundaries’ definition
- Failure and repair process identification
- Reliability data collection and risk factor exploration

Step 2: Reliability analysis of the selected item

- Reliability model selection
- Parameters’ estimation

Step 3: Spare-part management

- Spare-part estimation
- Inventory management

### 2.1 Context identification

Context identification of an item is essential in the spare-part estimation process using frailty modeling. This step consists of three sub-steps:

- Item identification and boundaries’ definition
- Failure and repair process identification
- Reliability data collection and risk factor exploration

In the first step, the system and its components should be identified. After that, its boundary needs to be determined for the selected component (item). The boundary will determine the required data and information needed. Moreover, it will prevent the identified item’s overlapping with adjacent items [27]. Two influential factors to be considered in setting the boundaries are the study level (fleet/machine/component) and the data constraints. In general, if the study level includes a fleet, each machine is considered as a component, each series as a subsystem, and the entire fleet as the system. If the study subject is a specific machine/item of equipment, it needs to be broken down into its components for further study [28]. Different methods, such as reliability block diagram, fault tree, reliability graph, and simulation, can be used to identify the item and set its boundaries. The selected item’s failure and repair process needs to be studied in the next step. The failure data collection methods can be divided into quantitative and qualitative approaches. The failure and repair data can be collected through documents, archived documents, visits and interviews, direct observations, joint observations, and artificial work [29]. Besides, the item’s operating conditions data should be collected and sorted for further analysis as risk factors. The data needed for reliability analysis, and especially spare-part management, include the type of failure, failure time, repair data, and their observed risk factors [30].

After data collection, the collected data should be checked for any heterogeneity in the next step. Among various methods proposed for the homogeneity test, the likelihood ratio test, Akaike information criterion (AIC), and the Bayesian information criterion (BIC) are standard tests for examining homogeneity in a specific database [31,32]. For example, under the assumption of Weibull distribution for failure data, the likelihood ratio will be as follows:

$$R=2\left(\ln\;L(\hat{\lambda },\hat{\beta },\hat{\eta },\hat{\theta })-lnL({\hat{\lambda }}_{0},{\hat{\beta }}_{0},{\hat{\eta }}_{0},0)\right)$$ (1)

where $\hat{\lambda }$ and $\hat{\beta }$ are the estimated parameters for Weibull distribution, $\hat{\eta }$ is the regression coefficient for observed risk factors, *and* $\hat{\theta }$ is the degree of heterogeneity due to the effect of unobserved risk factors.

### 2.2 Reliability analysis of item

For effective reliability analysis, the collected data’s best-fit model should be identified. In addition to the heterogeneity test, the time dependency of observed risk factors should be evaluated to select the appropriate model. The available time-dependency analysis models can be categorized into analytical and graphical approaches. Among the analytical models, the Schoenfeld Residuals test is one of the most widely used analytical tests [33–35]. In general, based on the nature of collected data, if there is an unobservable risk factor, the frailty model is appropriate; otherwise, the PHM and its extension can be used for reliability analysis. In the presence of *p*_*1*_ time-independent risk factors, *(z)*, *and p*_*2*_ time-dependent risk factors, (*z(t)*), the frailty model takes the following form [33,34]:

$$\lambda (t;z;z(t);\alpha )=\alpha \lambda_{0}(t)exp\left[\sum_{i=1}^{p_{1}}\eta_{i}z_{i}+\sum_{j=1}^{p_{2}}\delta_{j}z_{i}(t)\right]$$ (2)

where *λ*_*0*_ *(t)* is the baseline hazard rate, the function $exp[\sum_{i=1}^{p_{1}}\eta_{i}z_{i}+\sum_{j=1}^{p_{2}}\delta_{j}z_{i}(t)]$ captures the effect of observed risk factors, and *α* is the frailty. The *α > 1* are said to be frailer for reasons left unexplained by the observed risk factors and will have an increased risk of failure, while items with *α* < 1 are less frail; hence, given a specific observed risk factor pattern, they tend to be more reliable. If there is no effect from unobserved risk factors, then *α* = 1, and Eq (1) will reduce to the PHM [9]. As Eq (2) shows, frailty represents one or more unobserved risk factors’ cumulative effect. Given the relationship between the hazard rate and the reliability functions, it can be shown that the conditional reliability function, *R*(*t*;*z*;*z*(*t*)|*α*), can be written as [33,34]:

$$R(t;z;z(t)|\alpha )={\left\{R(t;z;z(t))\right\}}^{\alpha }$$ (3)

The unconditional (population) reliability function can then be estimated by integrating the unobservable *α*. If *α* has probability density function *g(α)*, then the population or unconditional reliability function is given by [33,34]:

$$R_{\theta }(t;z;z(t))=\int_{0}^{\infty }{\left\{R(t;z;z(t))\right\}}^{\alpha }g(\alpha )d\alpha$$ (4)

The relationship between the survival function and the hazard function still holds unconditional on α, and thus we can obtain the population hazard function, using [33,34]:

$$\lambda_{\theta }(t;z;z(t))=-\frac{d}{dt}R_{\theta }(t;z;z(t)){\left[R_{\theta }(t;z;z(t))\right]}^{-1}$$ (5)

The gamma distribution with the mean equal to one and variance *θ* is the most widely applied frailty distribution. The unconditional reliability function with gamma distribution unobserved risk factors is given by [33,34]:

Rθ(t;z;z(t))=[1−θln{R(t;z;z(t))}]−1θ (6)

In general, distribution should be selected to represent the baseline reliability. Baseline reliability represents a system’s reliability when there is no effect from observed and unobserved risk factors. The best-fit distribution for the baseline can be determined using a goodness-of-fit (GOF) test such as AIC and BIC criteria. These two criteria are based on the information and are utilized by classically comparing the maximum likelihood value to select the appropriate model. These two criteria are formulated as follows [31]:

AIC=−2×ln(likelihood)+2×k (7)

BIC=−2×ln(likelihood)+ln(N)×k (8)

where *k* indicates the number of estimated parameters, and *N* represents the number of observations (failures). The model with the smallest AIC and BIC values will be selected as the most appropriate choice in an appropriate model fitting. Further information on these two criteria can be found in [31,36].

After model selection, the reliability parameter should be estimated. Having the event times, (*t*_0*i*_, *t*_*i*_, *d*_*i*_), for *i* = 1,…,*n* with the *i*th observation corresponding to the time (*t*_0*i*_, *t*_*i*_], with either failure occurring at a time *t*_*i*_ (*d*_*i*_ = 1) or the failure time being right-censored at time *t*_*i*_ (*d*_*i*_ = 0); the likelihood function for failure data can be used for parameter estimation as follows:

$$LnL=ln\prod_{i=1}^{n}\frac{{\left\{R_{\theta i}(t_{i},z_{i},z_{i}(t)\right\}}^{1-d_{i}}{\left\{f_{\theta i}(t_{i},z_{i},z_{i}(t)\right\}}^{d_{i}}}{R_{\theta i}(t_{i},z_{i},z_{i}(t)}$$ (9)

where *f*_*θi*_ is the probability density function.

### 2.3 Spare-part management

This step includes the spare-part estimation and inventory management. After the selection of an appropriate model for reliability modeling of the selected item, the meantime to failure $\left(\overset{-}{T}\right)$ and the standard deviation of time to failure (σ(*t*,*z*;*z*(*t*)))) for the selected item can be calculated. These are the two main elements for estimating the required number of spare parts. Different mathematical models have been developed to estimate the required number of spare parts of repairable and non-repairable items. For example, the homogeneous Poisson process and renewal theory are two widely used mathematical models to estimate the required number of spare parts for non-repairable items. The homogeneous Poisson process is a particular state of the renewal process. Renewal theory is also used to estimate spare parts for parts with variable hazard rates. Based on the homogeneous Poisson process model, with this assumption that the replacements are made within the $\overset{-}{T}$, the mean number of failures (*M*_*t*_) can be estimated by renewability function as [5,10]:

Mt=tT−+(σ(t,z;z(t)))T−)2−12 (10)

where

T−=∫0∞tf(t)dt⇒f(t)=−R′(t)=−∫0∞tR′(t)dt=∫0∞R(t)dt=∫0∞([1−θln{R(t;z;z(t))}]−1θ)dt (11)

σ(t,z;z(t)))=∫0∞(t−T−)2f(t)dt⇒f(t)=−R′(t)=−∫0∞(t−T−)2R′(t)dt=2∫0∞tR(t)dt−T−2=(2∫0∞tR(t)dt−T−2)12=(2∫0∞t([1−θln{R(t;z;z(t))}]−1θ)dt−T−2)12 (12)

The mean number of failures for a specific period (typically on an annual basis), *M*(*t*), we can estimate the “Economic Order Quantity (EOQ)” and reorder point to eliminate the possibility of facing shortages. This EOQ can be calculated by [9]:

$$EOQ=\sqrt{\frac{2M_{t}S}{H}}$$ (13)

S is an order cost per purchase order, and H is the cost of storing a unit in stock for a year. Finally, the reorder point (Rp) should be estimated. Rp can be estimated based on the different scenarios where lead time and demand can be constant or varying. For example, under the assumption of constant lead time and variable demand, Rp can be estimated by:

$$Rp=Average\;demand\;during\;lead\;time+Safety\;stock=(\overset{-}{M_{t}}\times L)+(Z\times \sigma_{dLT})$$ (14)

where $\overset{-}{M_{t}}$ is average demand in time scale (day, week, or months) *L* is a lead time in same time scale Z is the number of standard deviation needed to achieve the defined cycle-service level and *σ*_*dLT*_ is the standard deviation of demand during lead time. It should be mentioned that Z value is the inverse of the standard normal cumulative distribution for a given service level. For more information see [37,38].

## 3. Case study

Golgohar iron mine is located 50 km southwest of Sirjan city. Currently, there are seven active sections in this mine. Section No. 1, with a definite reserve of 228 million tons and an annual extraction of 12 million tons, is one of these sections. In this section, most of the loading ore and waste rocks operations are performed by Caterpillar excavators. The production system is a series configuration; if the loading system fails, the entire extraction operation will stop. Hence, excavator spare-part planning is crucial to reduce excavator downtime and, consequently, stoppages in mining activity. Due to the nature of the rock and the mine’s operational condition, the excavator bucket nail fails frequently and needs to be changed regularly. In this study, the excavator bucket nail (EBN) is considered for further analysis.

Fig 1 shows that the failure mechanism and its associated risk factors should be identified after boundary selection. The reliability data for EBNs and their associated risk factors are collected from different sources, including daily reports, repair shop reports, meteorological reports, and interviews with specialists and experts. Table 1 shows the identified risk factors and their associated quantitative values. According to Table 1, working shifts (morning, evening, and night shifts), type of rock (high-density ores and tailings), and operator crew are defined as scaled risk factors, while the temperature (*z*_4_) and precipitation (*z*_5_) are considered continuous risk factors. In Table 2, a sample of the collected failure data and their associated risk factors are shown. According to this table, the first failure happened after 59.8 hours, during the night shift, while the operational condition temperature was 6.74^°^C. At the time of failure, there was precipitation (20.9 mm per hour), the excavator was working on tailings, and operator crew No. 3 was working with the machine.

**Table 1:** The classification of risk factors.

Risk Factors	Classified	Quantitative Values
**Shift (*z***_**1**_**)**	Morning	1
	Evening	2
	Night	3
**Type of Rock (*z***_**2**_**)**	Tailings	1
	Ore	2
**Operator Crew (z**_**3**_**)**	A	1
	B	2
	C	3
	D	4
**Temperature (*z***_**4**_**)**	Continuous risk factors	
**Precipitation (*z***_**5**_**)**	Continuous risk factors	

**Table 2:** Some of the TTF data associated with Caterpillar excavator risk failure factors.

Failure Number	Time to Failure (TTF)	Failure Status	Risk Factors				
			Shift	Temperature (C)	Precipitation (mm)	Type of Rock	Operating Group
**1**	59.8	0	3	6.74	20.9	1	3
**2**	14.28	0	1	2.59	11.07	2	2
**3**	26.19	1	2	11.24	19.13	1	1
**4**	26.48	1	3	12.45	18.18	1	1

In the next step, the collected reliability should be explored for the presence of unobserved heterogeneity, as well as time-dependency risk factors. In this study, the LR test in Eq (1) is used to evaluate the homogeneity of the data as below:

$$R=2\left(\ln\;L(\hat{\lambda },\hat{\beta },\hat{\eta },\hat{\theta })-lnL({\hat{\lambda }}_{0},{\hat{\beta }}_{0},{\hat{\eta }}_{0},0)\right)=23.56$$ (15)

R’s value for the excavator bucket nail is equal to 23.56, with a P-value equal to zero that indicates that the null hypothesis (absence of unobserved heterogeneity) should be rejected in favor of an alternative hypothesis. In other words, there are unobservable risk factors that need to be modeled in the reliability analysis. Hence, a frailty model can be used for reliability analysis. In the next stage, the risk factors’ time-dependency should be investigated, checked using the Schoenfeld residual test. The result of this test is shown in Table 3. As this table shows, the *p-value* for all risk factors is more than 5%. Hence, the null hypothesis, which is the time-dependency of risk factors, can be rejected. In other words, all risk factors can be considered time-independent risk factors.

**Table 3:** The P-value values for the estimation of the PH of risk factors.

Risk Factors	Ρ	χ_2_	D.F	P-value
**Shift**	0.005	0	1	0.92
**Temperature**	-0.075	2	1	0.13
**Precipitation**	0.001	0	1	0.98
**Type of Rock**	0.014	0	1	0.77
**Operational Group**	0.035	1	1	0.47
**Total Test**	-	3	5	0.73

**Fig 2:**
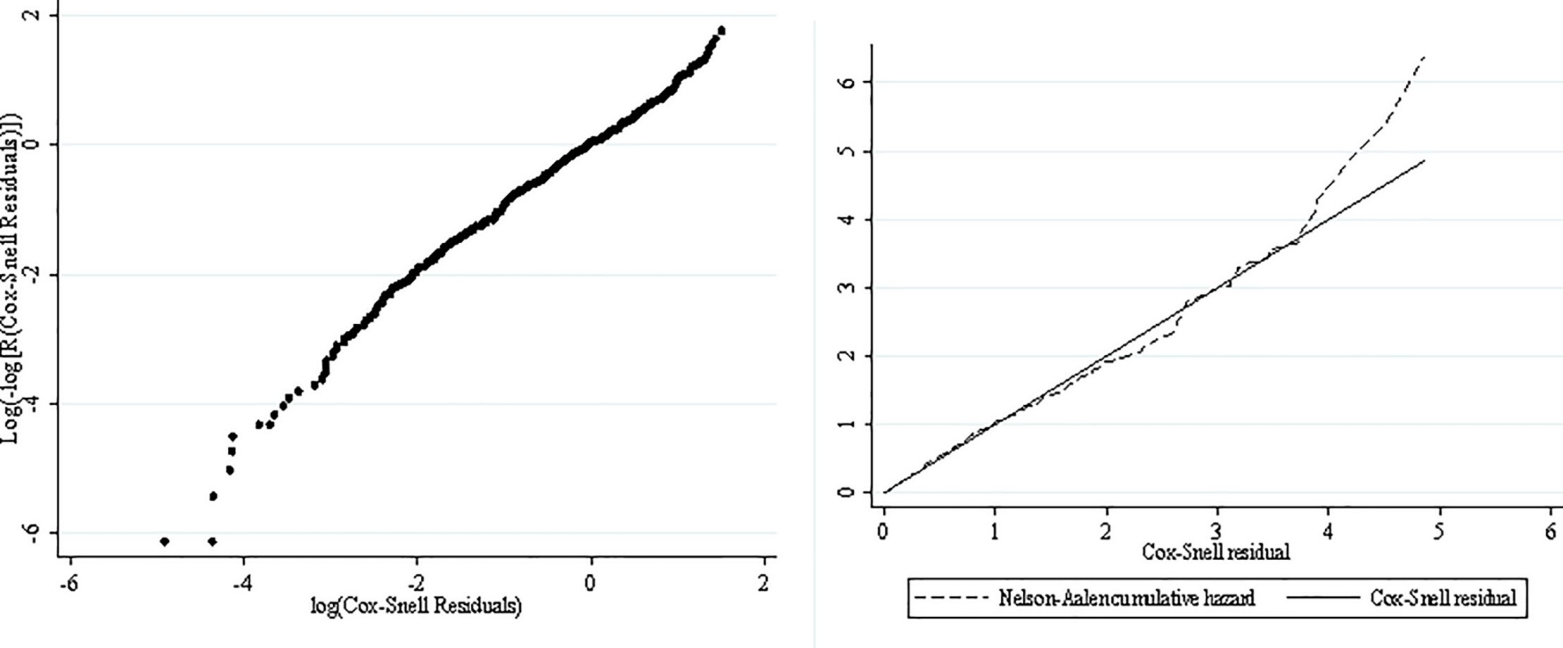
The Cox-Snell residual diagram of the exponential frailty model.

The best-fit distribution for the baseline hazard rate needs to be identified in the next step. In this analysis, the Weibull distribution and Exponential distribution are nominated to represent the *R*(*t*;*z*;*z*(*t*)). AIC and BIC statistics are used to determine the best-fit distribution. The results of AIC and BIC statistics are shown in Table 4. The frailty model with exponential baseline distribution (exponential frailty) has the lowest value and has the most appropriate fit among the nominated distributions. Furthermore, the Cox-Snell residual diagram is used to check the model’s goodness graphically. In this test, if the hazard function follows the 45-degree line, it can be concluded that exponential distribution is an appropriate model for the reliability modeling of the data. As Fig 2 shows, the hazard function follows the 45-degree line and, hence, the exponential distribution can be used to model the reliability data of the EBN. According to this model, the reliability function of the EBN can be written as:

Rθ(t;z)=[1−θln{(e−(tScale)shape)exp(∑j=1nωizi)}]−1θ=[1−θln{(e−(tβ)α)exp(∑j=1nωizi)}]−1θ (16)

where σ and β are shape and scale parameters of the exponential distribution, *ω*_*i*_ is the regression coefficient of risk factors, and *θ* is the heterogeneity degree.

**Table 4:** The GOF values of regression functions.

Model	AIC	BIC
**Weibull Frailty**	1527.6	1564.7
**Exponential Frailty**	1525.6	1558.6

Using the likelihood function, the shape and scale parameters of the exponential distribution, the coefficients of risk factors, and the frailty function’s heterogeneity value are calculated. The results are shown in Table 5.

**Table 5:** The Exponential frailty estimated parameters.

Risk Factors	Risk Factor Coefficients	Standard Error	η	p-value	95% Con. Interval	
**Shift**	0.016	0.075	0.22	0.825	-0.13	0.163
**Temperature**	-0.003	0.008	-0.43	0.67	-0.018	0.012
**Precipitation**	-0.005	0.006	-0.73	0.468	-0.017	0.008
**Type of Rock**	0.604	0.134	4.52	0	0.342	0.866
**Operational Group**	-0.084	0.056	-1.49	0.135	0.193	0.026
**Degree of heterogeneity**		θ = 0.297				
**Baseline parameters**		Shape Parameter:1; Scale Parameter: 97.232				

As shown in Table 5, only the type of rock has a *p-value* less than 5%, and the rest of the identified risk factors have a *p-value* greater than 5%. Hence, only the type of rock has a significant effect on the reliability performance of the EBN. Hence, the exponential-frailty reliability function of the EBN can be developed as:

Rθ(t;z)=[1−0.297×ln{(e−(t97.232)1)exp(0.604z2)}]−10.297 (17)

In the next step, the classical approach has been used to estimate the reliability of EBN. The classical approach is used to check the Frailty model’s result with the current practice. In the classical approach, the only variable is TTF. We nominate several distributions, including Weibull, lognormal, exponential. The result of GOF tests showed that the best fit distribution is the Exponential distribution with the parameters shown in Table 6.

**Table 6:** The Exponential classical estimated parameters.

	Coef.	Std. Error	Z	P>|z|	95% Confidence Interval	
**_cons**	-4.386	0.048	-90.740	0.000	-4.481	-4.291
**Lambda**	0.012	0.001	20.690	0.000	0.011	0.014

Based on the estimated parameters in Table 6, the reliability function of the EBN can be developed as:

$$R(t;z)=e^{-\frac{t}{\left(\frac{1}{0.012}\right)}}$$ (18)

**Fig 3:**
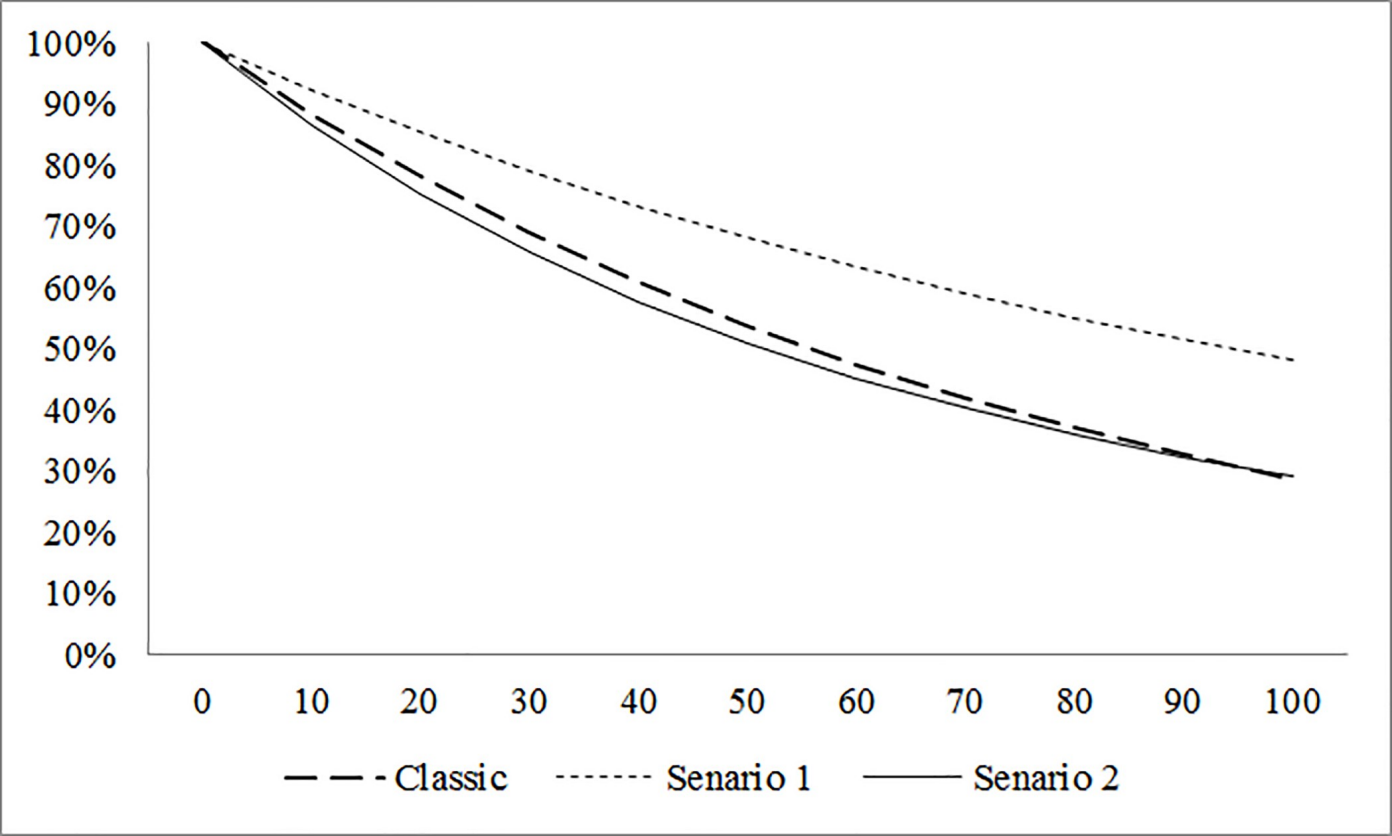
Reliability diagram of the classical and frailty exponential function for EBN.

Using Eq (17) and the defined value for the rock type in Table 1 (Tailings: 1 and Ore: 2), Fig 3 shows the calculated reliability graph for 100 hours of operation of the EBN while it works on ore rock (Scenario No. 1) and tailing rock (Scenario No. 2). Moreover, Eq (18) is used to plot the reliability of EBN without considering the observed and unobserved risk factors.

As this graph shows, the reliability of the EBN at the end of 50 hours of operation is 68.80% on ore rock and 50.90% on tailing rock. It can be concluded that more spare parts are needed while it is working on tail rocks. Moreover, it showed that the reliability of EBN is underestimated in the classical approach compared to ore rock and overestimated compared to tailing rock, which is estimated based on the frailty model.

Using the result of reliability analysis, the required number of spare parts can be estimated. As the selected item is non-repairable. Two parameters, σ(*t*,*z*;*z*(*t*))) and $\overset{-}{T}$ need to be estimated within the performance time interval, *t*. The numerical values of these two parameters, in Scenario No.1, can be calculated as:

T−=∫0∞R(t)dt⇒T−=∫0∞[1−0.297×ln{(e−(t97.232)1)(1.83)}]−10.297dt=75.59 (19)

σ(t,z;z(t)))=2∫0∞t[1−0.297×ln{(e−(t97.232)1)(1.83)}]−10.297dt−T−2=118.53 (20)

**Fig 4:**
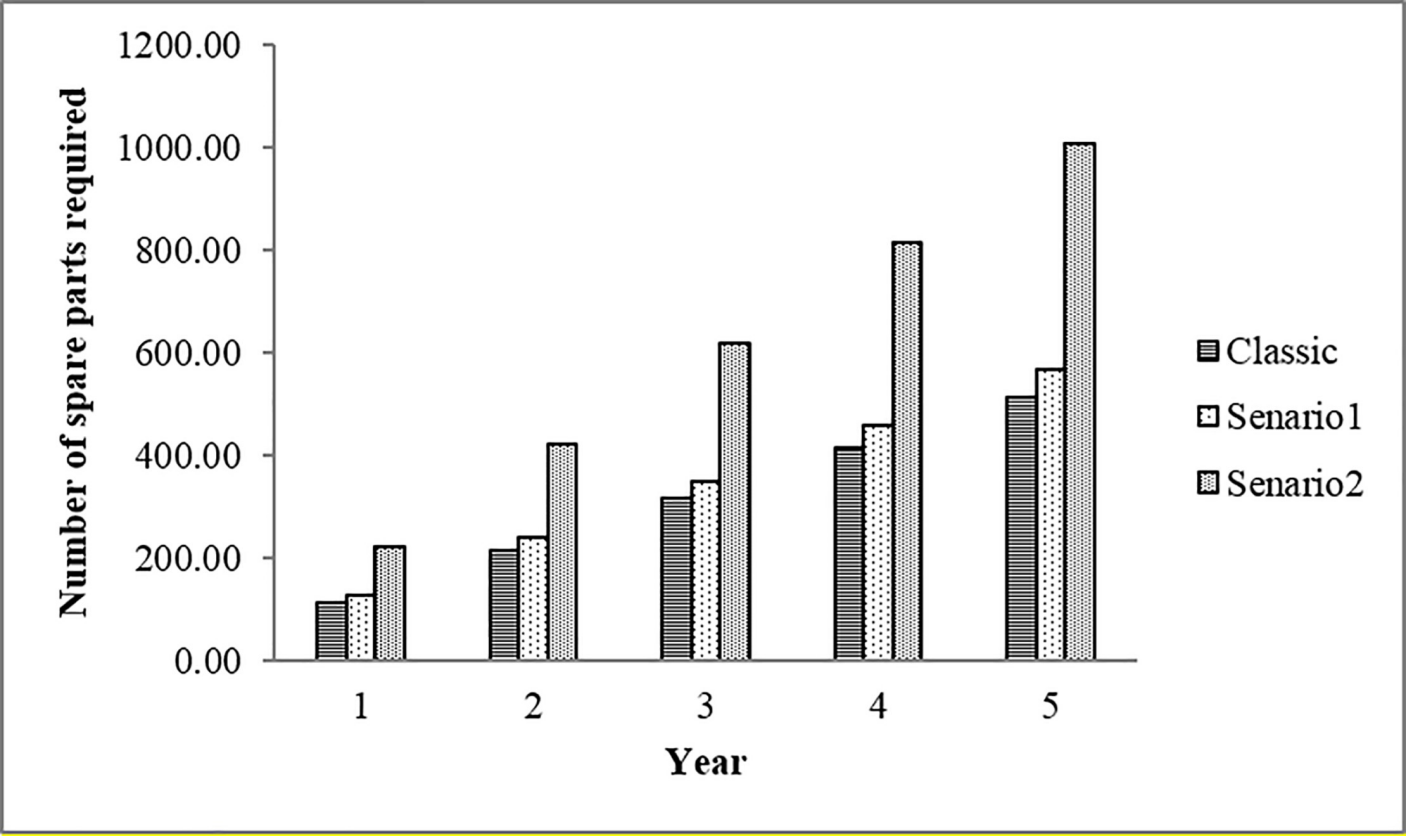
The number of EBN.

Having σ(*t*,*z*;*z*(*t*))) and $\overset{-}{T}$ operation time, the number of spare parts required (*M*_*t*_) can be calculated. Here, the estimation is carried out for 365 days, with an average daily operation of 21.5 hours. By considering the probability of a 5% shortage, the required number of spare parts is shown in Fig 4.

**Fig 5:**
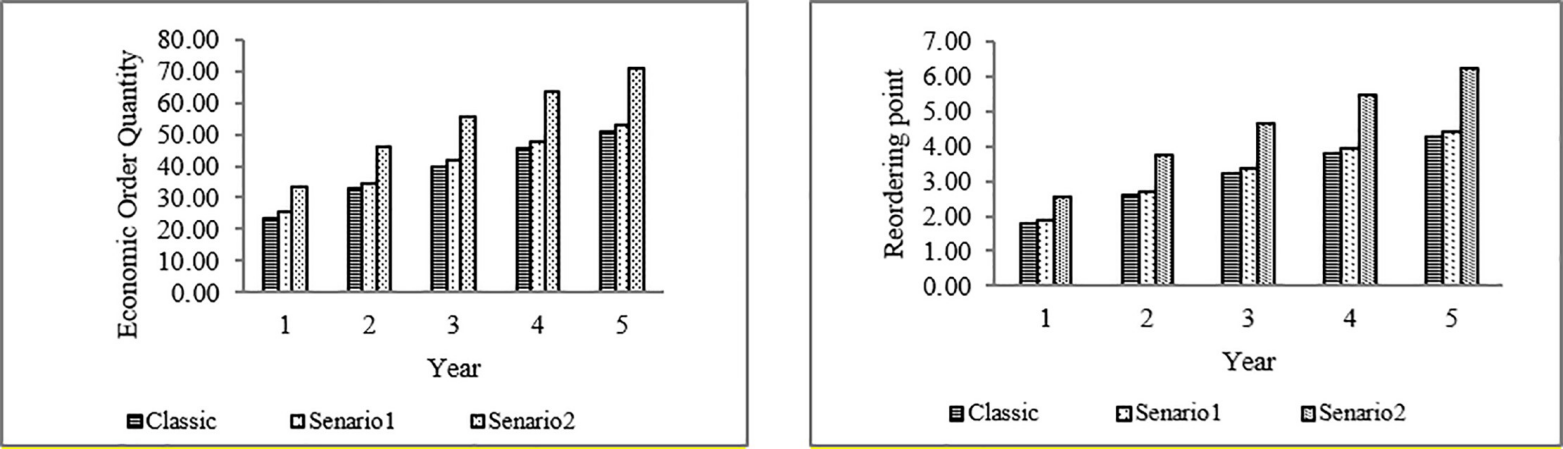
The amounts for re-order shipping and the economic order quantities of EBNs.

As Fig 4 shows, the excavator needs more spare parts when it works on tailing rock (Scenario No. 2). Moreover, the Frailty model’s obtained result is significantly different from the classical approach. Finally, by considering the equivalent cost of 2$ per nail, the order cost of 0.5$, the annual maintenance cost of 0.2$, with an average delay time of five days and a confidence level of 90%, the number of economic order quantities (EOQ) and the Reorder Point for the excavator bucket are calculated and shown in Fig 5. Reflected by the second scenario, if the warehouse level reaches 2.57 (in reality, 3), we have to order 33.28 (in reality, 33) nails.

## 3.1. Conclusion

Spare-part estimation is the main element of spare-part management. The effective spare-part prediction will eliminate unnecessary stoppage and avoid the high costs of delays in the supply of spare parts at the time of repairs. Moreover, it optimizes the inventory size and minimizes the inventory management costs. In this study, we developed a frailty-based approach for spare-part estimation, incorporating the effect of observed and unobserved risk factors. In this approach, firstly, sorting and classifying the time series data and risk factors. Then homogeneity test needs to be carried out to assess the presence or absence of unobservable risk factors. After that, the meantime to failure and the standard deviation of failure need to be evaluated.

In estimating these parameters, available studies mostly ignore the effect of unobserved risk factors. Hence, in this paper, we generalized the previous approach to calculate the values of mean time to failure and the standard deviation of failure in the presence of observed and unobserved risk factors. The homogeneity test results show that some unobservable risk factors affect the EBN reliability performance. Hence, the frailty model is used to isolate the effect of unobserved and observed risk factors. The spare-part estimation analysis showed that ignoring the unobserved risk factors can lead to unrealistic estimations. Based on the frailty model, 240.36 EBNs are needed for the excavator if it is going to work on ore rocks. However, ignoring the risk factors reduces this to 138.76, which is an unrealistic estimation and can cause an unplanned stoppage of the excavator due to the shortage of EBNs.

## Supporting information

S1 Data (XLSX)
